# Factors associated with facial melasma severity in Brazilian women: an internet-based survey^⋆^^⋆^

**DOI:** 10.1016/j.abd.2023.12.008

**Published:** 2024-08-08

**Authors:** Ana Flávia Teixeira de Abreu, Marina Oliveira Dias, Mayla Martins Conti Barbosa, Rebecca Perez de Amorim, Hélio Amante Miot, Ana Cláudia Cavalcante Espósito

**Affiliations:** a Department of Infectology, Dermatology, Imaging Diagnosis and Radiotherapy, Faculty of Medicine, Universidade Estadual Paulista, Botucatu, SP, Brazil

Dear Editor,

Melasma is a chronic, recurrent, and multifactorial hypermelanosis that mainly affects women of reproductive age and, although there are known triggers (e.g., exposure to solar radiation, pregnancy, hormonal therapy, hormonal contraceptives), no study has systematically investigated the factors associated with facial melasma severity.^1^, ^2^

The multifactorial nature of melasma severity requires a large sample to perform a robust multivariate analysis. In this context, internet-based surveys are the methodology that enables research requiring large samples, which are inaccessible in clinical practice.

The study aimed to explore factors associated with facial melasma severity in adult women in Brazil, based on a cross-sectional internet-based survey, which included women aged 18 to 60 years old, who reported facial melasma previously diagnosed by a dermatologist. Participants who reported other concomitant facial dermatoses or dermatoses related to photosensitivity were excluded.

The project was approved by the Ethics Committee (Counsel number 5,509,091) and the participants were invited to answer an online form consisting of items on clinical, demographic, and exposure-related data, in addition to the Pittsburgh Sleep Quality Index (PSQI) questionnaire and the HAD (Hospital Anxiety and Depression) scale. Finally, they pointed out on a facial map (Fig. 1) the areas affected by melasma, with the number of affected areas indicating disease extent. The map was validated in person with 51 women (correlation with the mMASI severity scale: rho = 0.94, p < 0.01). Sample availability and invitation to participate occurred through discussion groups about melasma on social networks. The study was conducted during the months of June to September 2022.

**Fig. 1:**
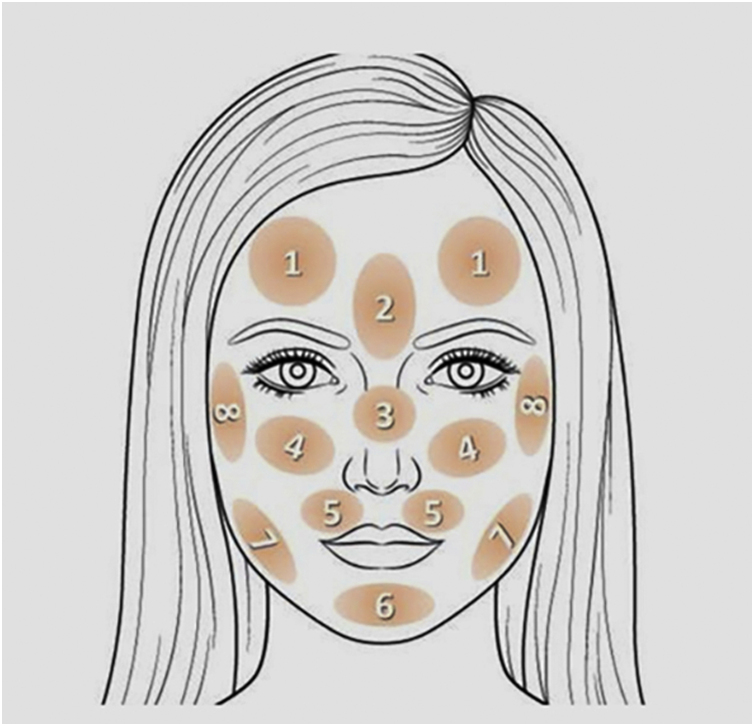
Validated facial map, used by participants to point out the regions affected by melasma on their faces.

The study-dependent variable was the severity of melasma, represented by the number of areas indicated on the facial map. The independent variables were demographic information, clinical data, demographics, occupational exposure, HAD, and PSQI.

The facial areas indicated on the facial map were evaluated by cluster analysis (Ward's method) and the covariates were tested for the number of topographies using a generalized linear model with Tweedie probability distribution (identity link). The size of the effect was estimated by the β coefficient of regression and statistical significance was defined as p-value < 0.05.

Of the 2,271 responses, 1,878 were considered valid due to diagnostic confirmation, duplications, or incomplete data. Table 1 highlights the main characteristics of the studied population. The predominance of women of childbearing age, self-declared white, who had more than one pregnancy and a high frequency of positive family history stands out. The high prevalence of anxiety disorders, depression and impaired sleep quality is also worth highlighting in this sample. The main worsening factors were sun exposure, heat exposure and psychological stress.

**Table 1:** Main clinical and demographic data of the 1,878 women with melasma, aged 18 to 65 years old.

Variables		Values	
Age (in years), mean (SD)		40.0	7.4
Self-declared skin color, n (%)			
	White	1398	74.4%
	Brown/Yellow	452	24.1%
	Black	28	1.5%
Number of pregnancies, n (%)			
	No pregnancies	682	36.3%
	One to three	1.125	59.9%
	More than three	71	3.8%
Hormonal pregnancy prevention, n (%)			
	Contraceptive/pregnancy	465	24.8%
	Menopause/hysterectomy	182	9.7%
	Implants/IUD with hormones	235	12.5%
Age at melasma onset (in years), mean (SD)		29,8	(6,5)
First-degree family member with melasma, n (%)		954	50,8%
Melasma extension score, median (p25-p75)		4	(2‒6)
Affected areas, n (%)			
	Forehead region	841	44.8%
	Region between the eyebrows?	407	21.7%
	Nasal region?	355	18.9%
	Malar region?	1399	74.5%
	Upper lip region?	602	32.1%
	Chin region?	209	11.1%
	Jaw region?	261	13.9%
	Temporal region?	733	39.0%
Lives in a polluted area, n (%)		319	17,0%
Current smoker, n (%)		70	3,7%
Sun-exposed, n (%)		32	1,7%
Practices sports exposed to the sun, n (%)		39	2,1%
Exposure to heat at work, n (%)		55	2,9%
Goes to the sauna, n (%)		9	0,5%
Use of prescription glasses, n (%)		794	42,3%
Adherence to photoprotector, n (%)		1553	82,7%
Comorbidities, n (%)			
	Thyroid disease	244	13.0%
	Migraine	362	19.3%
	Hypertension	111	5.9%
	Dyslipidemia	182	9.7%
	Diabetes	68	3.6%
Factor that triggered the melasma, n (%)			
	Sun exposure	1030	54.8%
	Contraceptive pill, injection or IUD	587	31.3%
	Pregnancy	560	29.8%
	Very intense psychological stress	382	20.3%
	Heat exposure	226	12.0%
	I don’t know	205	10,9%
	Facial hair removal	138	7,3%
	Skin procedure: peeling	129	6,9%
	Skin procedure: laser	115	6,1%
	Medication taken	52	2,8%
Melasma worsening factors, n (%)			
	Sun exposure	1408	75,0%
	Heat exposure	651	34,7%
	Psychological stress	460	24,5%
	I don’t know	192	10,2%
	Contraceptive pill, injection or IUD	187	10,0%
	Skin procedure: peeling/laser	133	7,1%
	Pregnancy	117	6,2%
HAD-A, mean (SD)		8,3	(4,0)
	HAD-A ≥8	1001	53,3%
HAD-D, mean (SD)		6,0	(3,7)
	HAD-D ≥8	609	32,4%
PSQI, mean (SD)		6,6	(3,5)
	PSQI >5	1084	57,7%

PSQI, Pittsburgh Sleep Quality Index; HAD-A, Hospital Anxiety Scale; HAD-D, Hospital Depression Scale; IUD, Intrauterine Device; SD, Standard Deviation.

According to the topographic distribution of facial melasma, three main cluster patterns were identified (Fig. 2): upper face, centro-facial and peripheral. According to the multivariate analysis (Table 2), more extensive melasma was identified in women with intermediate skin pigmentation, aged between 30 and 45 years old, who reported more than three pregnancies, who exposed themselves to the sun during work activities, who exposed themselves directly to heat, who had early onset of melasma, higher scores of anxiety, depression and impaired sleep quality. Adherence to sunscreen, however, showed a positive association with the severity of melasma, while practicing sports showed a negative association.

**Fig. 2:**
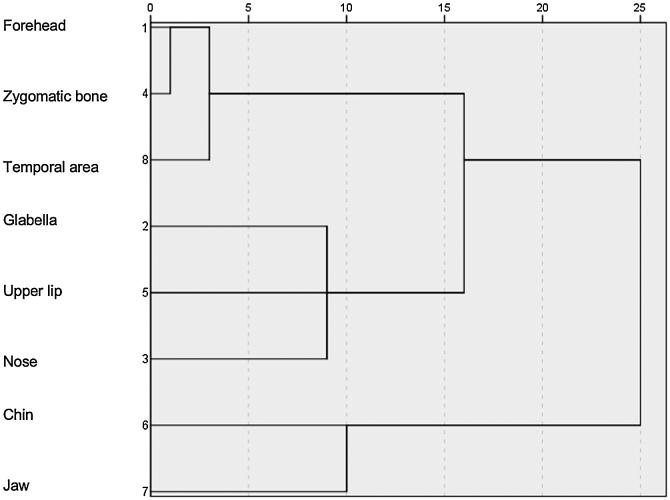
Cluster analysis of regions with melasma.

**Table 2:** Multivariate analysis of factors associated with melasma severity in adult women (n = 1,878).

Variables		β Coefficient	SE	p-value^a^
Self-declared skin color				
	White	‒	‒	‒
	Brown/Yellow	0.19	0.07	**0.011**
	Black	0.25	0.26	0.340
Age				
	<30 years	‒	‒	‒
	30‒45 years	0.27	0.11	**0.016**
	>45 years	0.22	0.14	0.123
Current smoking		−0,30	0.18	0.091
Air pollution in the region		−0,09	0.08	0.245
History of pregnancies				
	None	‒	‒	‒
	Between one and three	0.02	0.07	0.826
	More than three	0.35	0.18	**0.047**
Sun exposure at work		0,23	0.07	**0.001**
Sun exposure during sports practice		−0,18	0.07	**0.009**
Direct exposure to heat/sauna		0,22	0.09	**0.020**
First degree family history		0,14	0.06	**0.018**
Onset of melasma <30 years of age		0,30	0.06	**<0.001**
Daily adherence to sunscreen		0,18	0.08	**0.022**
Daily use of prescription glasses/lenses		0,00	0.06	0.901
Hormone therapy				
	None	‒	‒	‒
	Menopause	−0.25	0.12	**0.035**
	IUD with hormone	−0.03	0.09	0.764
	OC/Current pregnancy	0.09	0.08	0.260
HAD-D ≥ 8		0.15	0.07	**0.034**
PSQI > 5		0.22	0.06	**0.001**

SE, Standard Error; OC, Oral contraceptive; HAD-D, Hospital Depression Scale; PSQI, Pittsburgh Sleep Quality Index.

a adjusted p-value.

As the HAD-anxiety and HAD-depression values ​​were collinear (rho = 0.66; p < 0.01), they could not be included in the final model, and the one with the greatest statistical weight was chosen. However, both conditions were, when analyzed independently in the final model, associated with greater facial melasma severity (p < 0.05).

The clinical and demographic characteristics were similar to those found in other series of Brazilian women with melasma.^1^, ^2^, ^3^ The results of this study corroborate several previously mentioned aggravating factors, such as sun exposure, constitutional skin pigmentation, multiparity, genetic predisposition, and sexual hormones. Moreover, it reinforces the impact of disturbances of mental health, such as mood disorders and impaired sleep quality, as facial melasma aggravating factors.^4^

Affective disorders and sleep deprivation influence serum cortisol levels and increase systemic oxidative stress, substrates for the intensification of melanin pigmentation.^4^ Furthermore, a greater frequency of use of antidepressants and anxiolytics is observed in women with melasma, so this causality must be explored.^5^

High-dose infrared radiation can also induce melanogenesis, as it occurs in erythema *ab igne*. And, as in the present sample, a survey in India showed an association between melasma intensity and occupational exposure to heat.^6^, ^7^

Female sex hormones are known risk factors, which were corroborated in the present study by multiparity and greater severity in women who started melasma at an early age (<30 years), as opposed to those who were menopausal when the disease started.

Data regarding skin color in Latin American countries are predominantly self-reported, which may differ from phenotypic classification based on ethnicity, photoreactivity, or colorimetric aspects.^8^ In this sample, melasma was more intense in pigmented phenotypes; however, 74.4% of the participants reported being white, which may signal a possible social bias (due to greater access to internet), but also because melasma is more prevalent among individuals self-declared as white and brown, to the detriment of black people.

Similarly, adherence to sun-exposed physical activity was low (2%), hindering the inference of causality related to this factor. These data must be confirmed regarding the aspect of a sedentary lifestyle and melasma since several dermatoses are influenced by metabolic changes triggered by sports, and people with melasma may try to avoid sports in the sun.^9^

Among the participants, 24.3% reported photo-exposed leisure activities. In the last decade, there has been a population increase in photoexposure during resting time.^10^ In the present sample, the daily use of sunscreen was associated with greater melasma intensity. However, this information must represent a confounding variable, since more severe cases must lead to a greater search for treatment, in which photoprotection is essential.^9^

This study has limitations as it is not randomized and is based on invitation on social networks, which may generate social selection and adherence biases. However, a multivariate analysis was carried out and the variables were adjusted, with their β coefficients being estimated, thus minimizing the individual effect of disproportions. The different factors identified should be explored in randomized studies, with population sampling proportional to age and racial quotas, which more equally represent the Brazilian population, aiming to confirm these findings.

In conclusion, factors linked to genetics, sun and heat exposure, sexual hormones, affective comorbidities and sleep disorders were associated with facial melasma severity, which allows the development of pathophysiological hypotheses, therapeutic proposals and prevention measures.

## Financial support

None declared.

## Authors' contributions

Ana Flávia Teixeira de Abreu: Design and planning of the study, drafting and editing of the manuscript and approval of the final version of the manuscript.

Marina Oliveira Dias: Design and planning of the study, drafting and editing of the manuscript and approval of the final version of the manuscript.

Mayla Martins Conti Barbosa: Design and planning of the study, drafting and editing of the manuscript and approval of the final version of the manuscript.

Rebecca Perez de Amorim: Design and planning of the study, drafting and editing of the manuscript and approval of the final version of the manuscript.

Hélio Amante Miot: Design and planning of the study, drafting and editing of the manuscript and approval of the final version of the manuscript.

Ana Cláudia Cavalcante Espósito: Design and planning of the study, drafting and editing of the manuscript and approval of the final version of the manuscript.

## Conflicts of interest

None declared.
